# Draft genome sequence of *Achromobacter aegrifaciens* BAW48 isolated from arsenic-contaminated tubewell water in Bangladesh

**DOI:** 10.1128/mra.00097-24

**Published:** 2024-09-24

**Authors:** Anamica Hossain, M. Nazmul Hoque, Momtaz Zamila Bukharid, M. Anwar Hossain, Munawar Sultana

**Affiliations:** 1 Department of Microbiology, University of Dhaka, Dhaka, Bangladesh; 2 Molecular Biology and Bioinformatics Laboratory, Department of Gynaecology, Obstetrics and Reproductive Health, Bangabandhu Sheikh Mujibur Rahman Agricultural University, Gazipur, Bangladesh; 3 Jashore University of Science and Technology, Jashore, Bangladesh; SUNY College of Environmental Science and Forestry, Syracuse, New York, USA

**Keywords:** draft genome, *A. aegrifaciens*, arsenic (As), pollution, tubewell water

## Abstract

We report the draft genome of *Achromobacter aegrifaciens* strain BAW48, a bacterium with a genome size of 6,877,653 bp. This genome comprises gene clusters for arsenic conversion, such as arsenic resistance (*arsHCsO*), arsenite oxidation (*aioBA*), and arsenate reduction (*arsRCDAB*), along with genes for heavy metal and antibiotic resistance.

## ANNOUNCEMENT

Seventy-five million people in Bangladesh face chronic exposure to water containing>50 µg/L arsenic (As) (1), surpassing the WHO limit of 10 µg/L (2). *Achromobacter aegrifaciens* thrives in aerobic environments like freshwater, marine habitats, and soil (3). Close relatives of *A. aegrifaciens* BAW48 are capable of transforming arsenite to arsenate (4), and we therefore sequenced the genome of BAW48 to further explore this possibility.

*A. aegrifaciens* BAW48 was isolated from As-contaminated tubewell water samples, collected from the Bogura (24.85° N; 89.37° E) district of Bangladesh (1). In brief, the collected water sample (250 mL) underwent filtration using a sterile 0.22-µm cellulose-nitrate filter (Osmonics, USA). Bacteria from the filtrate were then enriched in 60 mL of minimal salt medium (MSM) agar plates containing 2 mM of NaAsO2 at 30°C for 7 days, and pure colonies were screened following previously published protocols (1). The genomic DNA from the BAW48 isolate was extracted from the pure colony by the FavorPrepTM Tissue Genomic DNA Extraction Mini Kit (FavorGen-Europe, Vienna), and purity and concentration of the extracted DNA were checked by NanoDrop 2000 UV–Vis Spectrophotometer (Thermo Fisher, USA). Libraries were generated from 1 ng of DNA using the Nextera DNA Flex Library Prep Kit (Illumina, USA), and whole-genome sequencing (WGS) was conducted using the Illumina NovaSeq PE150 sequencer (Illumina, San Diego, CA, USA) with a 2 × 250-bp protocol (5, 6). The paired-end raw reads (*n* = 81,960,888 bp) were trimmed using Trimmomatic v0.39 (7) to remove Illumina adapters, known Illumina artifacts and phiX, and quality-checked using FastQC v0.11.7 (8). Reads (*n* = 81,720,440) with phred scores > 20 were assembled using SPAdes v.3.9.0 (9), and quality of the assembled genome was checked using QUAST v.5.0.2 (10). Genome completeness was assessed through CheckM v1.2.2 using the *Achromobacter* CheckM marker set (11). The NCBI Prokaryotic Genome Annotation Pipeline v6.4 (12) was used for genome annotation. ResFinder 4.0 (13), VFDB (14), and RAST FIGfams v.70 (15) databases were used to predict antibiotic resistance genes (ARGs), virulence factors, and metabolic functions, respectively, in the draft genome. Default parameters were used for all software unless otherwise specified.

**Fig 1:**
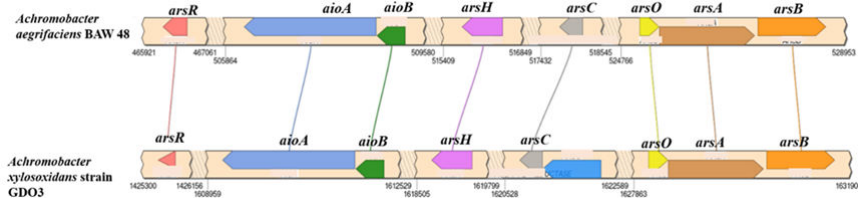
Arsenic transforming gene repertoire of the *Achromobacter aegrifaciens* strain BAW48. Arsenic (As) transforming gene clusters such as arsenite oxidizing *aio*BA, arsenate reducing *ars*RCDAB, and the monomethyl arsenite [MMA(III)] oxidizing *ars* resistance gene (*ars*HCsO) are depicted in the BAW48 genome against the reference genome of *Achromobacter aegrifaciens* strain GDO3. We used SimpleSynteny (http://www.SimpleSynteny.com), a web-based tool to generate this figure.

The draft genome of BAW48 had 45 x coverage and 98.11% completeness through CheckM that possessed 52 contigs. The annotated genome length, GC content, and N_50_ value were 6,877,653 bp, 65.5%, and 767,704, respectively. The BAW48 genome contained 352 subsystems 6,319 protein-coding genes, and 73 RNA genes. Three As transforming gene clusters such as arsenite oxidizing *aio*BA, arsenate reducing *ars*RCDAB, and monomethyl arsenite [MMA(III)] oxidizing *ars* resistance gene (*ars*HCsO) cluster were predicted in the BAW48 genome (Fig. 1). Our WGS data suggest that the BAW48 isolate is unique in genome organization and has potential as a bioremediating agent, especially for As detoxification yet not brought to attention.

## Data Availability

The whole genome of the ***Achromobacter aegrifaciens* BAW48** has been deposited at NCBI/GenBank under the accession number JAVCPK000000000, and the raw reads or SRA data can be cited under BioProject accession number PRJNA1004903. The version described in this paper is version JAVCPK000000000.1.

## References

[1] Diba F, Hoque MN, Rahman MS, Haque F, Rahman KMJ, Moniruzzaman M, Khan M, Hossain MA, Sultana M. 2023. Metagenomic and culture-dependent approaches unveil active microbial community and novel functional genes involved in arsenic mobilization and detoxification in groundwater. BMC Microbiol 23:241. doi:10.1186/s12866-023-02980-0 37648982 PMC10466822

[2] Rahaman MS, Mise N, Ichihara S. 2022. Arsenic contamination in food chain in Bangladesh: a review on health hazards, socioeconomic impacts and implications. Hyg Environ Health Adv 2:100004. doi:10.1016/j.heha.2022.100004

[3] Brenner DJ, Krieg NR, Staley JT, Garrity G. 2005. Bergey's manual of systematic bacteriology: volume two: the proteobacteria (part C). Springer.

[4] Diba F, Khan MZH, Uddin SZ, Istiaq A, Shuvo MSR, Ul Alam A, Hossain MA, Sultana M. 2021. Bioaccumulation and detoxification of trivalent arsenic by Achromobacter xylosoxidans BHW-15 and electrochemical detection of its transformation efficiency. Sci Rep 11:21312. doi:10.1038/s41598-021-00745-1 34716390 PMC8556249

[5] Hoque MN, Jerin S, Faisal GM, Das ZC, Islam T, Rahman A. 2023. Whole-genome sequence of multidrug-resistant Escherichia coli strains isolated from mice with mastitis. Microbiol Resour Announc 12:e0032023. doi:10.1128/mra.00320-23 37314348 PMC10353464

[6] Hoque MN, Moyna Z, Faisal GM, Das ZC, Islam T. 2023. Whole-genome sequence of multidrug-resistant Klebsiella pneumoniae MNH_G2C5, isolated from bovine clinical mastitis milk. Microbiol Resour Announc 12:e0007923. doi:10.1128/mra.00079-23 37093061 PMC10190633

[7] Bolger AM, Lohse M, Usadel B. 2014. Trimmomatic: a flexible trimmer for Illumina sequence data. Bioinformatics 30:2114–2120. doi:10.1093/bioinformatics/btu170 24695404 PMC4103590

[8] Andrews S. 2010. FastQC: a quality control tool for high throughput sequence data. Babraham Bioinformatics, Babraham Institute, Cambridge, United Kingdom.

[9] Bankevich A, Nurk S, Antipov D, Gurevich AA, Dvorkin M, Kulikov AS, Lesin VM, Nikolenko SI, Pham S, Prjibelski AD, Pyshkin AV, Sirotkin AV, Vyahhi N, Tesler G, Alekseyev MA, Pevzner PA. 2012. SPAdes: a new genome assembly algorithm and its applications to single-cell sequencing. J Comput Biol 19:455–477. doi:10.1089/cmb.2012.0021 22506599 PMC3342519

[10] Gurevich A, Saveliev V, Vyahhi N, Tesler G. 2013. QUAST: quality assessment tool for genome assemblies. Bioinformatics 29:1072–1075. doi:10.1093/bioinformatics/btt086 23422339 PMC3624806

[11] Parks DH, Imelfort M, Skennerton CT, Hugenholtz P, Tyson GW. 2015. CheckM: assessing the quality of microbial genomes recovered from isolates, single cells, and metagenomes. Genome Res 25:1043–1055. doi:10.1101/gr.186072.114 25977477 PMC4484387

[12] Tatusova T, DiCuccio M, Badretdin A, Chetvernin V, Nawrocki EP, Zaslavsky L, Lomsadze A, Pruitt KD, Borodovsky M, Ostell J. 2016. NCBI prokaryotic genome annotation pipeline. Nucleic Acids Res 44:6614–6624. doi:10.1093/nar/gkw569 27342282 PMC5001611

[13] Bortolaia V, Kaas RS, Ruppe E, Roberts MC, Schwarz S, Cattoir V, Philippon A, Allesoe RL, Rebelo AR, Florensa AF, et al.. 2020. ResFinder 4.0 for predictions of phenotypes from genotypes. J Antimicrob Chemother 75:3491–3500. doi:10.1093/jac/dkaa345 32780112 PMC7662176

[14] Liu B, Zheng D, Zhou S, Chen L, Yang J. 2022. VFDB 2022: a general classification scheme for bacterial virulence factors. Nucleic Acids Res 50:D912–D917. doi:10.1093/nar/gkab1107 34850947 PMC8728188

[15] Overbeek R, Olson R, Pusch GD, Olsen GJ, Davis JJ, Disz T, Edwards RA, Gerdes S, Parrello B, Shukla M, Vonstein V, Wattam AR, Xia F, Stevens R. 2014. The SEED and the rapid annotation of microbial genomes using subsystems technology (RAST). Nucleic Acids Res 42:D206–D214. doi:10.1093/nar/gkt1226 24293654 PMC3965101

